# Clinical and genetic characteristics in lymphoma patients with a second solid malignancy

**DOI:** 10.3389/fonc.2023.1152290

**Published:** 2023-07-21

**Authors:** Di Zhou, Leng Han, Chanjuan Jin, Lintao Bi

**Affiliations:** Department of Hematology and Oncology, China-Japan Union Hospital of Jilin University, Changchun, Jilin, China

**Keywords:** multiple primary malignancies, mutation, lymphoma, FANC, germline mutation, somatic mutation

## Abstract

Diagnosis and treatment of multiple primary malignancies are becoming a new challenge in clinical practice worldwide. The present study aimed to characterize the clinical and genetic features of multiple primary malignancies in patients with synchronous or metachronous lymphoma and another solid tumor. We retrospectively analyzed 11 cases with lymphoma and another solid tumor. The germline mutations in plasma cell-free DNA samples and somatic mutations in lymphoma and solid tumor tissue samples were identified using targeted next-generation sequencing. In the 11 lymphoma patients, the most common type of concurrent solid tumor was colon adenocarcinoma (case 3, 5, 9 11) followed by papillary thyroid carcinoma (case 1, 7, 10). Metachronous lymphoma and solid tumor in 6 patients were treated with corresponding standard therapy asynchronously. Chemotherapy for colon adenocarcinoma during the interval of lymphoma chemotherapy led to excellent outcome in two patients. Immediate chemotherapy for lymphoma plus elective surgery for synchronous papillary thyroid carcinoma also yielded good prognosis in two patients with synchronous double primaries. Interestingly, we found that 10 of 11 patients with lymphoma and another solid tumor harbored germline mutations in Fanconi anemia complementation group (FANC) genes, including FANCI, FANCA, FANCG, FANCL, FANCD1, FANCF, FANCJ, and FANCS. In summary, comprehensive study of the clinical and genetic features of patients with multiple primary malignancies may improve diagnosis and treatment in the future. Mutations in FANC genes might be a predisposition to tumorigenesis of lymphoma patients with a second solid malignancy.

## Introduction

During the last three decades, substantial progress has been made in the prevention, early diagnosis, and treatment of malignancies. Advances in early detection and effective treatment have significantly improved overall survival in patients with malignant tumors, such as proteomics used in ovarian cancer (1). The five-year survival rate for all cancers is nearly 40.5% in China and 68% the United States (2, 3). As of January 2019, there were approximately 16.9 million cancer survivors in the United States (3). With the aging of the population, the number of cancer survivors is projected to be greater than 22.1 million by early 2030 (3). One of the new health challenges that cancer survivors may face is developing a second primary cancer. Patients with two or more histologically unrelated malignancies arising independently are diagnosed with multiple primary malignancies (4, 5).

Multiple primary malignancies are defined as more than one synchronous or metachronous cancers in which the second primary malignant tumor is diagnosed within or after 6 months of the primary one, respectively (4). Epidemiological studies reported that the frequency of multiple primary malignancies varies from 2% to 17% (6–9). The second primary cancer usually complicates the situation, and diagnosis and treatment for multiple primary malignancies have become a new challenge in clinical practice (10). Genetic and environmental factors may contribute to the development of multiple primary malignancies. Except for hereditary multiple cancer syndromes, such as Li-Fraumeni syndrome, the genetic variations responsible for the development of sporadic multiple primary malignancies are largely unknown. All survivors of lymphoma, including Hodgkin lymphoma (HL) and non-Hodgkin lymphoma (NHL), have an increased risk of developing a secondary cancer (11, 12). It has been traditionally thought that high-dose extensive radiation and chemotherapy with alkylating agents are linked with a higher risk of secondary cancer (13). Radiation is now given locally in lower doses. Moreover, second primary malignancies can emerge synchronously with lymphoma and thus are unlikely secondary to the treatment of lymphoma (14). The mechanisms underlying the development of lymphoma and other solid malignancies in the same individual are still unclear, and it is unknown whether genetic susceptibility contributes to the pathogenesis of such multiple malignancies.

The present study aimed to characterize the clinical and genetic features of multiple primary malignancies in lymphoma patients. We summarized the diagnosis, treatment, and prognosis of 11 patients with synchronous or metachronous lymphoma and another solid tumor. We also determined and compared the germline and somatic genetic mutations in both tumor tissues/cells.

## Materials and methods

### Patients and data collection

This study enrolled 11 lymphoma patients who were diagnosed with multiple primary malignancies and treated at the China-Japan Union Hospital of Jilin University. The diagnosis of lymphoma and other solid tumors was confirmed by biopsy. Multiple primary malignancies were defined as two or more histologically unrelated, independent primary malignancies in one individual and were diagnosed using the criteria proposed by the Surveillance Epidemiology and End Results (SEER) project and the International Association of Cancer Registries and International Agency for Research on Cancer (IACR/IARC). Synchronous multiple tumors were diagnosed in an interval of less than 6 months, while metachronous tumors were diagnosed in an interval of more than 6 months. Only patients with lymphoma (HL or NHL) and another solid cancer were included in the present study. Patients with more than two primary malignancies were excluded from this study. The demographic and clinical data were obtained by medical record review. The Institutional Review Board of the China-Japan Union Hospital of Jilin University (Jilin, China) approved the study protocol (#20220628020), and the project has therefore been performed in accordance with the ethical standards laid down in the 1964 Declaration of Helsinki and its later amendments. All procedures followed were in accordance with the ethical standards of the responsible committee on human experimentation (institutional and national) and with the Helsinki Declaration of 1975, as revised in 2008 (5). All involved patients provided written informed consent.

### Targeted next-generation sequencing

Next-generation sequencing was carried out to detect germline mutations in plasma cell-free DNA samples and somatic mutations in lymphoma tissue and solid tumor tissue samples as previously described (15). Briefly, genomic DNA was extracted from the above-mentioned samples using the Maxwell FFPE DNA kit following the manufacturer’s instructions. The DNA libraries were prepared using the KAPA Hyper Prep Kit (KAPA Biosystems, Wilmington, MA, USA). Target enrichment was performed using the probes targeting 437 cancer-related genes (Geneseeq Prime panel) for detections of germline mutations and somatic mutations of solid tumors and using the probes targeting 475 key genes in hematologic malignancies (Hemasalus) for detections of somatic mutations in lymphoma tissues. The target-enriched libraries were amplified by quantitative polymerase chain reaction using the KAPA Library Quantification kit (KAPA Biosystems) and sequenced on a HiSeq 4000 sequencing platform (Illumina, San Diego, CA, USA). Sequencing data were processed and analyzed as previously described (15, 16). Briefly, germline variations were identified by comparing plasma cell-free DNA sequences with the hg19 reference genome, while somatic mutations were identified by comparing variations in tumor tissues with germline variations. Variants identified from plasma cell-free DNA sequencing with allele fractions greater than 50% were considered as germline variations which were also present in lymphoma tissues. Germline variations were compared with Fanconi Anemia Mutation Database (LOVD v.3.0) and NIH Single-Nucleotide Polymorphism database. Variations with less than 1% of frequency in the population were considered as mutations.

## Results

Among the 11 patients with an average age of 57 years old, four of them had a history of heavy smoking or drinking, and only two patients had a family history of cancer. The detailed demographic characteristics and medical history of the patients were summarized in **Table 1**.

**Table 1:** Demographic characteristics and medical history of patients.

Case	Age	Gender	BMI (Kg/m^2^)	Smoking	Drinking	Family History
1	56	F	20.6	No	No	No
2	53	M	24.6	No	No	No
3	61	M	20.4	Heavy	Heavy	No
4	60	M	22.1	Heavy	Heavy	No
5	64	M	20.3	Heavy	No	No
6	58	M	25.4	Heavy	Heavy	Brother died of gastric cancer
7	46	F	19.9	No	No	Father died of lung cancer
8	61	F	28.6	No	No	No
9	71	M	28.8	Light	Light	No
10	27	F	18.0	No	Light	No
11	66	M	21.2	No	No	No

BMI, body mass index.

Six of the 11 lymphoma patients suffered diffuse large B cell lymphoma (DLBCL), and all were non-germinal center B-cell (non-GCB) subtype. Five patients had synchronous while six had metachronous multiple primary malignancies. The interval of metachronous multiple primary malignancies ranged from 19 to 96 months. Lymphoma and colon adenocarcinoma are the most common combination of synchronous multiple primary malignancies, which occurred in three patients. Two patients had HL while nine had NHL with the most common type being non-GCB DLBCL in our patients. In the 11 lymphoma patients, the most common type of concurrent solid tumor was colon adenocarcinoma followed by papillary thyroid carcinoma. The diagnosis and staging of lymphoma and the concurrent solid tumor were listed in **Table 2**.

**Table 2:** Clinical and tumor characteristics of patients.

Case	Type	Interval (months)	Lymphoma		Second solid tumor	
			Diagnosis	Stage	Diagnosis	Stage
1	Meta	19	AITL	IVB	PTC	II
2	Meta	80	HL	IIA	NSCLC	IV
3	Syn	–	DLBCL	IV	CRC	II
4	Meta	68	DLBCL	IIA	Laryngeal cancer	III
5	Meta	84	DLBCL	IVB	CRC	II
6	Meta	96	FL	IV	SQCLC	IV
7	Syn	–	DLBCL	IV	PTC	I
8	Meta	72	DLBCL	IIEA	NSCLC	IV
9	Syn	–	HCL	N/A	CRC	I
10	Syn	–	HL	IIA	PTC	III
11	Syn	–	DLBCL	IIA	CRC	IIIB

Meta, metachronous; Syn, synchronous; AITL, angioimmunoblastic T-cell lymphoma; HL, Hodgkin lymphoma; DLBCL, Diffuse large B-cell lymphoma; FL, follicular lymphoma; HCL, hairy cell leukemia; PTC, papillary thyroid carcinoma; NSCLC, non-small cell lung cancer; CRC, colorectal cancer; SQCLC, squamous cell lung cancer. "-" means no interval.

Patients with HL were treated with ABVD (adriamycin, bleomycin, vinblastine, dacarbazine) chemotherapy while the majority of patients with NHL were treated with CHOP (cyclophosphamide, doxorubicin, vincristine, and prednisolone)-based regimes (**Table 3**). The second solid tumors were treated with surgery, radiation, or chemotherapy. Two patients succumbed to DLBCL relapse and progression, and one patient died from side effects of chemotherapy more than 12 months after the diagnosis of their first malignancy, whereas other patients are still alive without cancer or under ongoing treatments. In patients with metachronous multiple primary malignancies, their lymphoma and another solid tumor were treated asynchronously. The treatment of synchronous lymphoma and another solid tumor is challenging. For the third case with synchronous DLBCL and colon adenocarcinoma, the patient was taking capecitabine orally for colon cancer while receiving RCVP (rituximab, cyclophosphamide, vincristine, prednisone) chemotherapy for DLBCL. The patient had intermittent hematochezia during the treatment and eventually succumbed to the disease. For the seventh and tenth cases with synchronous DLBCL and papillary thyroid carcinoma, the two patients received surgery for thyroid cancer after complete remission of DLBCL with R-CHOP (rituximab, cyclophosphamide, doxorubicin, vincristine, and prednisolone)-chemotherapy or after complete remission of HL with ABVD chemotherapy. For the ninth case with synchronous hairy cell leukemia and colon adenocarcinoma, the patient received surgery for colon cancer during the interval between targeted therapies with rituximab for hairy cell leukemia. Similarly, the eleventh patient with synchronous DLBCL and colon adenocarcinoma received surgery for colon cancer during the interval between chemotherapies with the R-CHOP regimes.

**Table 3:** Treatment and outcome of patients.

Case	Treatment		Outcome	OS (months)
	Lymphoma	Second solid tumor		
1	CHOPE + HSCT	Surgery	AITL relapsed, In chemotherapy	>47
2	ABVD	Bevacizumab, Pemetrexed, Carboplatin	Died from side effects of chemotherapy	85
3	RCVP	Capecitabine	Died from DLBCL relapse	14
4	CHOP	Surgery + Radiation	No relapse	>148
5	R-CHOP	Surgery	Died of DLBCL progression	91
6	Radiation, Rituximab, Lenalidomide	Radiation	In radiation and chemotherapy	>112
7	R-CHOP	Surgery	Complete remission	>16
8	R-CHOP	Osimertinib	In chemotherapy for lung cancer	>88
9	Rituximab, Bendamostine	Surgery	Rituximab maintenance	>16
10	ABVD + Radiation	Surgery	Complete remission	>16
11	R-CHOP	Surgery	No relapse	>11

CHOPE, cyclophosphamide, doxorubicin, vincristine, prednisone, and etoposide; ABVD, adriamycin, bleomycin, vinblastine, and dacarbazine; RCVP, rituximab, cyclophosphamide, vincristine, and prednisone; CHOP, cyclophosphamide, doxorubicin, vincristine, and prednisolone; R-CHOP, rituximab, cyclophosphamide, doxorubicin, vincristine, and prednisolone; OS, overall survival.

Ten of 11 patients had germline mutations in genes belonging to the Fanconi anemia complementation group (FANC), including *FANCI*, *FANCA*, *FANCG*, *FANCL*, *FANCD1*, *FANCF*, *FANCJ*, and *FANCS* (**Table 4**), although none of the patients had clinical manifestations of Fanconi anemia or its pathologic mutations. Other common mutations were found in the genes of *POLD1*, *ATM*, *EXT2*, and *TSC1*.

**Table 4:** Germline mutations of patients.

Case	Germline mutations
1	*FANCI* (missense, c.2875C>T, p.R959W, 55.7%), *FANCA* (missense, c.3031C>T, p.R1011C, 58.4%), *CDH1* (missense, c.546A>C, p.K182N, 57.2%), *PMS2* (missense, c.598G>A, p.V200I, 56.3%), *ERCC5* (missense, c.1873G>A, p.E625K, 55.9%), *PTCH1* (missense, c.1058A>G, p.K353R, 54.3%), *RHBDF2* (missense, c.325C>T, p.R109C, 59.0%)
2	*FANCI* (frameshift, c.36_37dup, p.T13Kfs*13, 53.1%), *TSC2* (missense, c.5312C>T, p.P1771L, 74.8%), *CHEK2* (missense, c.497A>G, p.Y166C, 53.3%)
3	*FANCG* (missense, c.1298G>A, p.R433Q, 52.0%), *FANCL* (missense, c.670A>G, p.T224A, 58.4%), *FANCD1* (missense, c.1211A>T, p.N404I, 54.0%), *POLE* (missense, c.1205G>A, p.C402Y, 50.7%), *KIT* (missense, c.910A>G, p.T304A, 50.0%)
4	*FANCF* (missense, c.184C>A, p.L62M, 58.0%), *POLD1* (missense, c.2198A>G, p.Q733R, 58.1%), *RHBDF2* (missense, c.1841A>T, p.E614V, 55.4%)
5	*FANCD1* (missense, c.10028A>C, p.E3343A, 57.5%), *BTK* (missense, c.1177G>A, p.G393R, 100%), *CDH1* (splice site, c.1566-7C>T, 55.4%)
6	*FANCI* (missense, c.1298A>T, p.H433L, 44.7%), *ATM* (splice site, c.5675-4T>A, 64.5%), *RECQL4* (missense, c.1115G>C, p.R372T, 58.0%)
7	*FANCI* (missense, c.284T>A, p.L95Q, 57.7%), *TP53* (missense, c.91G>A, p.V31I, 58.0%), *EXT2* (nonsense, c.941T>A, p.L314*, 52.2%), *RECQL4* (missense, c.1936C>T, p.R646C, 51.5%)
8	*FANCI* (missense, c.2875C>T, p.R959W, 54.3%), *ATM* (missense, c.8495G>A, p.R2832H, 53.0%), *EXT2* (missense, c.1471G>A, p.A491T, 57.2%), *LHCGR* (missense, c.1184G>A, p.R395H, 53.3%)
9	*FANCJ* (missense, c.587A>G, p.N196S, 54.1%), *ERCC2* (missense, c.2086T>C, p.W696R, 53.6%), *DENND1A* (missense, c.1660T>C, p.W554R, 51.0%), *NTHL1* (missense, c.349C>A, p.P117T, 50.3%), *RET* (deletion, c.1760_1761delinsAA, 58.1%)
10	*FANCS* (missense, c.2491T>G, p.Y831D, 59.0%), *POLD1* (splice site, c.1138-3C>T, 56.5%)
11	*TSC1* (missense, c.250G>A, p.A84T, 56.2%), *MYD88* (missense, c.65G>C, p.G22A, 57.2%), *MSH6* (missense, c.211A>C, p.N71H, 57.8%)

The percentage values in the parentheses stand for allele frequency.

In terms of somatic mutations, none of the patients had identical genetic variation profiles between their lymphoma and the other solid tumor. However, there were different somatic mutations in common genes in lymphoma and solid tumor tissues in the same patient (**Table 5**). For example, mutations in the *TET2* and KDR genes were identified in both angioimmunoblastic T-cell lymphoma and her papillary thyroid carcinoma tissues (**Table 5**). Somatic mutations in the common genes between lymphoma and solid tumor were also found in cases 7, 9, and 11 (**Table 5**).

**Table 5:** Somatic mutations in the tumor tissue of patients.

Case		Somatic mutations
1	AITL	*TET2, KDR, ECT2L*
	PTC	*TET2, KDR*
2	Lymphocyte-rich HL	–
	NSCLC	*TP53, SMARCA4, YAP1, ROS1*
3	DLBCL	*NAT1, IRF4, TP53, BTG2*
	CRC	*ERCC1, MTHFR, NQO1, GSTM1, CDK12*
4	DLBCL	*DPYD, ERCC1, GSTP1, MTHFR, NQO1, TYMS, XRCC1*
	Laryngeal cancer	*PIK3CA, ZNF217, CDKN2A, TP53*
5	DLBCL	*CTNNB1, RNF43*
	CRC	*DPYD, GSTP1, MTHFR, NQO1*
6	FL	*DICER1*
	SQCLC	*TP53, RAF1, RHOA*
7	DLBCL	*CDKN2A*
	PTC	*CDKN2A, CDA, DPYD, ERCC1, MTHFR, NQO1, TYMS*
8	DLBCL	*PRDM1, TP53, CD70, ARID1B, BTG2*
	NSCLC	*CDA, MTHFR, NQO1, TPMT*
9	HCL	*DPYD*
	CRC	*DPYD, ARAF*
10	Nodular sclerosis HL	–
	PTC	*DPYD, ERCC1, MTHFR, NQO1, TPMT, UGT1A1*
11	DLBCL	*ATRX, SGK1, DDX3X, CD70, DTX1, PIM1, TBL1XR1*
	CRC	*ATRX, SGK1, EPHA3*

AITL, angioimmunoblastic T-cell lymphoma; HL, Hodgkin lymphoma; DLBCL, Diffuse large B-cell lymphoma; FL, follicular lymphoma; HCL, hairy cell leukemia; PTC, papillary thyroid carcinoma; NSCLC, non-small cell lung cancer; CRC, colorectal cancer; SQCLC, squamous cell lung cancer. "-" means no mutation.

## Discussion

In the present study, we reported the demographic, clinical, and genetic characteristics of 11 lymphoma patients with a synchronous or metachronous second solid malignancy. We found that the coexistence of DLBCL and colon adenocarcinoma are common in patients with multiple primary malignancies. Synchronous lymphoma and solid tumor could be treated simultaneously or sequentially. Interestingly, we found that most patients with multiple primary malignancies had germline mutations in genes of the FANC family. Although genetic variation profiles of lymphoma and concurrent solid tumor were not identical in any patient, there were common somatic mutations in lymphoma and solid tumor tissues in the same patient.

In the two categories of multiple primary malignancies, synchronous multiple primaries are less common than metachronous tumors. According to previous observational studies, adenocarcinomas and squamous cell carcinomas were the most common types in multiple primary malignancies, while hematological malignancies such as lymphomas were the least frequent type (17). Therefore, synchronous combination of lymphoma and other solid carcinomas are considered as a rare condition (14, 18–21). A few cases of synchronous double primary tumors of DLBCL and digestive carcinoma have been reported as rare case reports (22–24). In our five cases of synchronous double primary malignancies, two patients had synchronous DLBCL and colon adenocarcinoma. The coexistence of DLBCL and colon adenocarcinoma has also been found in metachronous double primary malignancies in the present study. Interestingly, all six patients with DLBCL had the non-GCB subtype, suggesting that the non-GCB subtype may be more likely involved in multiple primary malignancies. The gastrointestinal tract is the most common location for the extranodal involvement of NHL, accounting for 20-40% of all extranodal disease and 10-15% of all NHL, with stomach being the most common site of involvement (60-75%) followed by the small bowel, ileum, cecum, colon, and rectum (25). However, primary colonic lymphoma is a rare malignancy (26). prevalence of GI in lymphoma is relatively common Therefore, it is challenging to differentiate coexistent colon adenocarcinoma from colonic involvement in patients with lymphoma. Comprehensive analyses of clinical features, image studies, and pathologic findings could be helpful for accurate diagnosis (18). Biopsy and pathological examinations are crucial for the confirmation of secondary malignancy in lymphoma patients with suspicious or clinically atypical lesions.

Metachronous multiple primary malignancies are usually treated separately and asynchronously. There is no standard treatment for synchronous lymphoma and solid tumors, and the choice of treatment could be difficult to make. Previous studies suggest that the malignancy that primarily affects the patient’s prognosis and outcome should be treated as a priority in patients with synchronous lymphoma and solid tumors (27). For instance, papillary thyroid carcinoma is usually less aggressive than most types of lymphoma. Specifically, DLBCL, which is considered as an aggressive lymphoma and being fatal without treatment, should be treated immediately after diagnosed. In our two patients with synchronous lymphoma and papillary thyroid carcinoma, lymphomas (DLBCL and HL) were treated first with chemotherapy while papillary thyroid carcinoma was treated later with elective surgery. Both patients achieved complete remission and have been tumor-free for 16 months so far. Their excellent outcome suggests that chemotherapy plus elective surgery should be recommended to patients with synchronous lymphoma and papillary thyroid carcinoma. Besides the types of malignancies, we should also consider the cancer staging. The present study involved three patients with synchronous lymphoma and colon adenocarcinoma. The two patients with relatively early-stage lymphoma received surgery for colon cancer during the interval between chemotherapies for lymphoma, resulting in a good prognosis and outcome. However, the patient with synchronous stage IV DLBCL and stage II colon adenocarcinoma died from DLBCL relapse and progression despite simultaneous chemotherapy for both malignancies. A previous case series study of patients with synchronous lymphoma and digestive system carcinoma reported that neoadjuvant chemotherapy for controlling colon carcinoma growth and metastasis during the interval of chemotherapy for lymphoma should be considered as this strategy has acceptable tolerance and low toxicity (18).

The etiology and pathogenetic mechanisms for multiple primary malignancies are largely unknown. Genetic, lifestyle, hormonal, and environmental factors may contribute to the development of multiple cancers in the same patient. Except for hereditary cancer syndromes, increasing evidence demonstrate that genetic mutations play an important role in sporadic multiple primary malignancies (28). A study reported that 21% of patients with multiple primary tumors had pathogenic or likely pathogenic mutations (29). One of the important findings of the present study was that most of our patients with double lymphoma and solid tumor had germline mutations in FANC genes. FANC proteins are involved in DNA inter-strand cross-link repair and maintenance of chromosome stability (30). Homozygous germline mutations in 22 FANC genes lead to Fanconi anemia while heterozygous mutations in FANC genes significantly increase malignancy susceptibility without causing Fanconi anemia (30). We have compared the identified FANC gene variations with Fanconi Anemia Mutation Database and confirmed that all the variations we found are not pathogenic mutations which cause Fanconi anemia and that their clinical significances are uncertain. The incidence of germline mutations in homologous recombination repair genes is 11–33% among men with metastatic castration resistant prostate cancer, and the most common DNA damage repair aberration in prostate cancer is FANCD1 (also known as BRCA2), followed by FANCA (31). Although germline mutations in FANC genes have been reported to increase risk for developing both hematological and solid cancers (32–34), their roles in tumorigenesis of multiple primary malignancies are largely unknown. A previous case study demonstrated that a germline *FANCA* mutation (p.Q405*) might be a predisposition to double primary malignancies of thyroid cancer gastric adenocarcinoma (35). These findings suggest that *FANCA* mutations may be a risk factor for multiple primary malignancies. One of the limitations of the present study is that it is difficult to confirm if the identified mutations in FANC genes are pathological mutations, which needs further investigation. In addition, the mutations identified in second generation sequencing in the present study need to be validated using polymerase chain reaction or Sanger sequencing in the future.

In the present study, somatic mutations in different tumor tissues in the same patient were not identical. Different mutations in common genes were identified in solid tumors and lymphoma tissues in a few patients. The role of these mutations in the development of multiple primary malignancies is unclear. However, identifying common genes in multiple primary malignancy tissues might help to find out potential targets for immunochemotherapy with monoclonal antibodies.

Taken together, the present study characterized the clinical and genetic features of patients with lymphoma and a second primary solid tumor. Mutations in FANC genes are associated with multiple primary malignancies, however, their causative role in the tumorigenesis of multiple primaries warrants further investigation.

## Data availability statement

The original contributions presented in the study are included in the article/**Supplementary Material**, further inquiries can be directed to the corresponding author.

## Ethics statement

The studies involving human participants were reviewed and approved by The Institutional Review Board of the China-Japan Union Hospital of Jilin University (Jilin, China), (#20220628020). The patients/participants provided their written informed consent to participate in this study.

## Author contributions

DZ, LH and CJ collected the data, performed the experiments, analyzed the data, authored or reviewed drafts of the paper, and approved the final draft. LB conceived and designed the experiments, prepared tables, authored or reviewed drafts of the paper, and approved the final draft. All authors contributed to the article and approved the submitted version.
